# Cell Kinetics in the Adult Neurogenic Niche and Impact of Diet-Induced Accelerated Aging

**DOI:** 10.1523/JNEUROSCI.2730-18.2019

**Published:** 2019-04-10

**Authors:** Alexander J. Stankiewicz, Farzad Mortazavi, Peter V. Kharchenko, Erin M. McGowan, Vasili Kharchenko, Irina V. Zhdanova

**Affiliations:** ^1^Department of Preclinical Research, BioChron LLC, Worcester, Massachusetts 01605,; ^2^Department of Anatomy and Neurobiology, Boston University School of Medicine, Boston, Massachusetts 02118,; ^3^Department of BioMedical Informatics, Harvard Medical School, Boston, Massachusetts 02115,; ^4^Harvard Stem Cell Institute, Cambridge, Massachusetts 02138,; ^5^Institute for Theoretical Atomic, Molecular & Optical Physics, Harvard University, Cambridge, Massachusetts 02138, and; ^6^Department of Physics, University of Connecticut, Storrs, Connecticut 06269

**Keywords:** adult neurogenesis, aging, cell kinetics, mathematical modeling, neurogenesis, zebrafish

## Abstract

Neurogenesis in the adult brain, a powerful mechanism for neuronal plasticity and brain repair, is altered by aging and pathological conditions, including metabolic disorders. The search for mechanisms and therapeutic solutions to alter neurogenesis requires understanding of cell kinetics within neurogenic niches using a high-throughput quantitative approach. The challenge is in the dynamic nature of the process and multiple cell types involved, each having several potential modes of division or cell fate. Here we show that cell kinetics can be revealed through a combination of the BrdU/EdU pulse-chase, based on the circadian pattern of DNA replication, and a differential equations model that describes time-dependent cell densities. The model is validated through the analysis of cell kinetics in the cerebellar neurogenic niche of normal young adult male zebrafish, with cells quantified in 2D (sections), and with neuronal fate and reactivation of stem cells confirmed in 3D whole-brain images (CLARITY). We then reveal complex alterations in cell kinetics associated with accelerated aging due to chronic high caloric intake. Low activity of neuronal stem cells in this condition persists 2 months after reverting to normal diet, and is accompanied by overproduction of transient amplifying cells, their accelerated cell death, and slow migration of postmitotic progeny. This combined experimental and mathematical approach should allow for relatively high-throughput analysis of early signs of pathological and age-related changes in neurogenesis, evaluation of specific therapeutic targets, and drug efficacy.

**SIGNIFICANCE STATEMENT** Understanding normal cell kinetics of adult neurogenesis and the type of cells affected by a pathological process is needed to develop effective prophylactic and therapeutic measures directed at specific cell targets. Complex time-dependent mechanisms involved in the kinetics of multiple cell types require a combination of experimental and mathematical modeling approaches. This study demonstrates such a combined approach by comparing normal neurogenesis with that altered by diet-induced accelerated aging in adult zebrafish.

## Introduction

Adult neurogenesis is a dynamic and highly controlled process that adds new functional neurons to a mature CNS (for review, see Bond et al., 2015). Changes in cell kinetics in a neurogenic niche can interfere with normal maintenance of brain tissue and prevent compensatory mechanisms from counteracting neurodegeneration, brain repair after trauma, or stroke. Such changes can range from increased quiescence or cell death to alternative modes of division, altered migration, or inability to incorporate into preexisting neural networks. Finding ways to promote adult neurogenesis through prophylactics or treatment requires understanding of which cells and at which stage of a disease are at the core of the problem, and thus could serve as an effective drug target. A combination of high-throughput experimental techniques, animal models with active neurogenesis, and mathematical modeling can reveal the specific changes in cell kinetics and identify those factors that interfere with adult neurogenesis.

Neurogenesis declines with age in all vertebrate species studied, including fish (Edelmann et al., 2013), rodents (Seki and Arai, 1995; Kuhn et al., 1996; Kempermann et al., 1998), nonhuman primates (Gould et al., 1999), and humans (Eriksson et al., 1998; Spalding et al., 2013; Bergmann et al., 2015). This area of research is of great significance, but of great uncertainty as well. Genetically determined differences in constitutive or reactive neurogenesis may, in part, explain high interindividual variability in cognitive dysfunctions with age, including neurodegenerative disorders, such as Alzheimer's and Parkinson's diseases. However, more research is needed to test these hypotheses, in view of other diverse factors, including environmental agents, stress or altered circadian rhythmicity facilitating brain aging, and increasing probability of disease states (Guarente, 2014; Chin-Chan et al., 2015; Musiek et al., 2015; Liguori et al., 2018).

Metabolic abnormalities, including those associated with diabetes or obesity, appear to have some of the most powerful effects on the aging process (Thorpe and Ferraro, 2004; Haslam and James, 2005; Barbagallo and Dominguez, 2014; Bonomini et al., 2015). Hyperglycemia can alter neurogenesis (Dorsemans et al., 2017a) and contribute to the development of neurodegeneration and neurodegenerative diseases (Cai, 2013). Our recent study implicates a chronic life-long high caloric intake (HCI) in leading young adult zebrafish to develop a premature aging phenotype (Stankiewicz et al., 2017). Apart from the shortened lifespan, these animals develop early scoliosis, sleep abnormalities, and compromised circadian patterns of behavior. Importantly, accelerated aging in these fish is associated with a remarkably low cell proliferation in the brain. To better understand this phenomenon and develop a relatively high-throughput methodology of characterizing cell kinetics within neurogenic niches of an adult brain under normal and pathological conditions, we combined the experimental and mathematical model approaches.

Here we show an orderly cell kinetics pattern of adult neurogenesis in young healthy zebrafish brain and its alterations in HCI fish. Those include high day-to-day variability in cell proliferation, low numbers of both neuronal stem cells (NSCs), which divide infrequently, and transient neuronal progenitor cells (NPCs), which make several consecutive divisions. Additionally, HCI leads to changes in the predominant mode of NPC division and their short lifespan, with slow migration of their postmitotic progeny. Together, these results demonstrate that chronic HCI interferes with adult neurogenesis on several levels, and that mathematical modeling can provide needed insights into normal neurogenesis and changes associated with a pathological process, and predict their outcomes.

## Materials and Methods

### 

#### 

##### Animals.

Adult male zebrafish (*Danio rerio*, WT AB strain), 12 ± 1 months old, were maintained on a 14 h light/10 h dark (14:10 LD) cycle, at 28°C, in 3 L tanks of a multitank system (Aquaneering), as per standard practices (Whitmore et al., 2000). Larval diet consisted of *ad libitum* live food of *paramecium* and Type L saltwater rotifers (*Brachionus plicatilis*). At 20 d post-fertilization (dpf), fish were divided into two treatment groups (>100 fish each, 10 fish per 3 L tank), with one treatment group receiving a regular diet (Control) and another a high calorie intake (HCI) diet, as per our earlier report (Stankiewicz et al., 2017). The Control animals were fed twice-a-day, at zeitgeber times (ZT) 1 and 8 (ZT0 = lights on time), with Gemma-300 pellets (Skretting) and supplemental live feed of *Artemia salina nauplii* (brine shrimp). Total weight of daily food available to each animal was equal to ∼1.7% of body weight, with brine shrimp constituting ∼20% of total food received. The age-matched HCI fish were maintained on the same feeding schedule, except for receiving higher amounts of Gemma-300 pellets, at ∼5% body weight per day. Although measuring the exact amount of food consumed by each fish was not possible under these group housing conditions, the time animals spent in active feeding following food administration was documented in both groups and, on average, was 30% longer for HCI fish (data not shown). At the age of 10 months (±1), ∼2 months before brain sample collection, all fish were moved to the Control diet, to avoid acute effects of different caloric intake. All animal procedures were performed in accordance with the Institutional Animal Care and Use Committee.

##### Nutritional value of feed: live brine shrimp and Gemma-300.

Brine shrimp nauplii contain 37%–71% protein, 12%–30% lipid, 11%–23% carbohydrate, and 4%–21% ash. The length of an average nauplius is 450 μm. Gemma-300 is ∼300 μm in size and contains 59% protein, 14% lipid (oil), 14% ash, 0.2% fiber, and 1.3% phosphorus.

##### Pulse-chase protocol, using BrdU and 5-ethynyl-2′-deoxyuridine (EdU).

Each fish, in both Control and HCI groups, received one exposure to BrdU (pulse) and one exposure to EdU (chase). The difference between the fish was in the number of days (1–15) elapsing between these two exposures to different thymidine analogs. The protocol was developed based on the preliminary data showing equal efficacy of BrdU and EdU to label the same S-phase cells, when available in parallel. This was tested by injecting EdU (i.p.) and immediately placing animals into a tank containing BrdU solution, followed by sample collection 2 h later. The time of exposure, ZT9–11, was chosen based on the time of the peak in the number of S-phase cells in zebrafish brain, which captures the majority of replicating cells on a given day (Akle et al., 2017).

**Figure 1.**
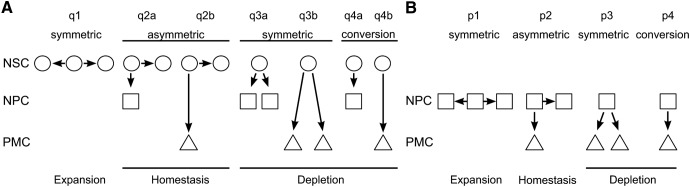
Different modes of cell division or conversion for NSCs and transient NPCs can lead to expansion, homeostasis, or depletion of their pool. ***A***, Modes of division/conversion for NSCs (circle). ***B***, Modes of division/conversion for transient NPCs (square). Postmitotic daughter cells (PMCs, triangle). For NSCs and NPCs, *q* and *p* are the probabilities of the mode of division, respectively.

On day 0, groups of six 1-year-old Control and HCI animals were transferred from their regular 3 L housing tanks into 1 L treatment tanks and immersed for 2 h in fish water containing BrdU solution (6.5 mm). After 2 h, fish were washed out in flow-through fish water and returned on to the recirculating housing system, where they remained for one or several days. On each of the following days, at ZT9, fish of one of the Control and one of the HCI groups were injected (i.p.) with EdU (50 μg/g in 1× PB solution, pH 7.4, 10 μl volume) and killed 2 h later, at ZT11. The Control and HCI groups received EdU on day 1, day 2, or day 3 after the initial BrdU exposure, one group per day (see Fig. 1). Because of the increased day-to-day variability in the number of cells in S-phase in HCI animals, compared with Control (as per the preliminary experiments), additional groups of HCI fish also received EdU on day 4 or day 5. In a separate set of experiments, involving both Control and HCI animals, an interval between BrdU and EdU exposure was increased to 15 d, to assess the survival of BrdU-labeled cells and their acquisition of neuronal fate.

Notably, exposure to the initial BrdU pulse via immersion allowed us to avoid stress that might affect cell kinetics over the following days. For the same reason, the EdU chase injection was conducted only shortly (2 h) before sample collection, when the S-phase was already at daily peak. Immersion in EdU was ruled out due to high reagent cost.

##### Immunohistochemistry in sectioned brains.

Fish were killed with an overdose of MS222 (200 mg/L), and heads were fixed overnight in 4% PFA in PBS at 4°C. Brains were then dissected out, cryoprotected in 30% sucrose/PBS, placed in embedding solution (OCT), and stored at −80°C, until cut in series. Coronal 20 μm sections collected systematically into 3 matched series were prepared using a cryostat (Microm HM505E) and placed onto Fisherbrand Superfrost Plus slides (Thermo Fisher Scientific), then stored at −80°C, until processed. At that time, one of the three matched series slides was removed from storage and thawed for immunohistochemistry. The sections were washed in 0.1 m PBS, and antigen retrieval was performed. The sections were incubated in 50% formamide/50% 2× SSC at 65°C for 2 h, and acid, 2 m HCl at 37°C for 30 min, and then in 0.1 m boric acid, pH 8.5, for 10 min at room temperature (RT). Following a PBS wash, the slides were outlined using a hydrophobic pen to minimize use of reagent. Approximately 150 μl of solution of mouse anti-BrdU antibody directly conjugated to AlexaFluor-555 (Invitrogen, 1:200; in 0.1 m KPBS + 0.4% Triton X-100) was added to each slide, and those were stored in a slidebox at 4°C overnight. After washing in PBS, sections were incubated in ∼175 μl of Click-iT reaction mixture from the Click-iT EdU AlexaFluor-488 Flow Cytometry Assay (Invitrogen). The mixture was added to the slides within 15 min of preparation, and they were incubated inside the slidebox for 30 min. After rinsing in PBS, slides were mounted using Vectashield DAPI mounting medium (Vector Laboratories) and sealed.

No difference in the uptake of BrdU or EdU was observed between the Control and HCI animals following the concurrent exposure to these thymidine analogs (2 h, ZT9–11), with individual cells labeled by BrdU or EdU displaying similar intensity of fluorescence in HCI and Control animals.

##### Whole-brain clearing to visualize cell proliferation and morphology in 3 dimensions.

Fish were killed with an overdose of MS222 (200 mg/L), and heads were fixed overnight in 4% PFA in PBS at 4°C. Brains were then dissected out, cryoprotected in 30% sucrose/PBS, placed in embedding solution (OCT), and stored at −80°C. For CLARITY, brains were gradually thawed by moving them from −80°C to −20°C, and then to 4°C. They were then washed at RT in 0.05 m TBS, pH 7.4, 2 × 15 min in 15 ml polypropylene conical tubes. Brains were then transferred into glass vials to prevent adherence of brains and incubated at 37°C for 2 h in CLARITY solution, 200 mm SDS containing 20 mm lithium hydroxide monohydrate, pH 9.0. Vials were gently agitated on a tissue rocker over 5–7 d at RT, until brains were completely transparent. The brains were then washed in 0.05 m TBS 2 × 12 h and then stained.

##### BrdU, EdU, and HuC/D antigen labeling in CLARITY-processed whole brains.

Brains were first incubated in 50% formamide/50% 2× SSC at 65°C for 1 h, then in acid, 2 m HCl, at 37°C for 15 min, and finally in 0.1 m boric acid, pH 8.5, for 5 min at RT. Following a 3 × 5 min TBS washes, the brains were incubated for 12 h overnight in a 500 μl solution of Click-iT reaction mixture from the Click-iT EdU AlexaFluor-488 Flow Cytometry Assay (Invitrogen) at RT, with vigorous agitation on a tissue rocker. Mouse anti-BrdU antibody directly conjugated to AlexaFluor-555 (Invitrogen, 1:200) and primary anti-HuC/D mouse IgG2B monoclonal 16AII (Invitrogen, 1:200) was added to the 500 μl of Click-iT reaction mixture and incubated in 4°C for 5 d. Brains were washed in TBS 2 × 30 min and incubated for 12 h overnight in secondary AlexaFluor-633 goat anti-mouse IgG2B (Invitrogen, 1:200) in TBS at RT. The brains were washed in TBS 2 × 12 h and stored in TBS at 4°C.

##### Image acquisition of cleared whole brains.

CLARITY-processed whole brains provide for 3D visualization of adult neurogenesis in zebrafish brain (Lindsey et al., 2018). To improve the refractive index for confocal imaging, we incubated the brains for 10–15 min in 80% glycerol solution. They were then carefully mounted between 2 glass-bottom wells. Using BluTack, each brain was positioned with the dorsal surface of the brain facing up. Brains were kept between the clear-bottom glass in a solution of 20% glycerol in 0.05 m TBS to avoid their drying out during imaging. Brains were imaged on a TCS SP8 confocal microscope with motorized stage (Leica Microsystems), using HC FLUOTAR L 25× VISIR, a water-immersion objective with high numerical aperture (0.95 NA), and a large free working distance. The Leica Microsystems native software was used for postprocessing data reconstruction. To count cells, the part of the *z* stack corresponding to a structure containing a neurogenic niche (e.g., cerebellum, hypothalamus, habenula) was isolated using the Bitplane Imaris program and saved as an individual file. For quantification of cells positive for BrdU, EdU, BrdU/EdU, and BrdU/HuC/D, the 3D object counter in Fiji was used, with each channel quantified individually. Colocalization was confirmed manually, based on *x*, *y*, *z* coordinates of each colabeled cell. The false-positive counts were eliminated based on voxel volumes (pixel^3^).

##### Microscopy, cell counts, and statistical analysis in sectioned brains.

Confocal microscopy was performed with an LSM 710 (Carl Zeiss) using the Observer Z1 inverted microscope. Using Zen software, images were taken with a 20× objective. To minimize crosstalk between channels in multicolored tissue, sequential image acquisition was performed. The labeled cells were counted in the whole cerebellum and, separately, within the cerebellar neurogenic niche, from the rostral end of the valvula cerebelli to the caudal end of corpus cerebelli (Grandel et al., 2006; Kaslin et al., 2009). The cerebellar neurogenic niches within the molecular layer of the medial division of the valvula cerebelli (Vam_mol_), lateral division of the valvula cerebelli (Val_mol_), and the corpus cerebelli (CCe_mol_) were differentiated from the migration zones of the greater cerebellum. Those within the granular layers of the medial the valvula cerebelli (Vam_gra_), lateral division of the valvula cerebelli (Val_gra_), and corpus cerebelli (CCe_gra_) were based on their distinct histological and anatomical differences (Adolf et al., 2006). The number of cells stained for BrdU and EdU was quantified in one of three matched series containing seven coronal 20 μm cerebellar sections per brain, five or six brains per condition (Control vs HCI), using the Volocity software 6.3 automated counting algorithm (PerkinElmer Improvision). Before this, extensive validation of the technique through comparison with manual cell counts was conducted by two independent experimenters, both blind to the treatment condition. For all quantifications, the values obtained for each of the seven sections were totaled to obtain a value per brain. To account for interindividual difference in brain size, the data were adjusted for brain volume, which was estimated based on the images of individual brain sections (ImageJ) and using the Cavalieri principle (Prakash et al., 1994), as reported previously (Akle et al., 2017). The effects of treatment day (i.e., chase interval) for each label (BrdU, EdU, BrdU/EdU) were assessed by an unpaired two-tailed Student's *t* tests for Control and HCI conditions, with *p* < 0.05 considered significant.

##### Mathematical modeling of cell kinetics in neurogenic niche.

To provide quantitative assessment of the time-dependent kinetics of proliferating cells in adult zebrafish brain, we developed a mathematical model describing quasi-periodic processes of the NSC and NPC division, postmitotic cell (PMC) generation, and their migration from the neurogenic niche to periphery. The general model captures different possible modes of NSC and NPC divisions (see Fig. 1*A*,*B*), using differential equations to describe time-dependent cell densities. The analytical solutions of the proposed kinetics equations can be used to perform parametric analysis of normal neurogenic processes and their alterations with aging and disease, or modified by environmental factors and drugs.

##### General formulation.

The master equations for the cell densities are given by the following expressions:

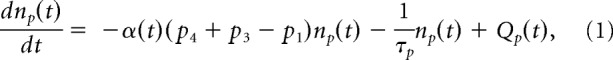


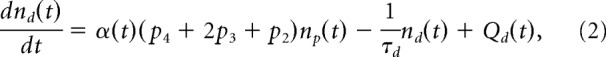
 where *n_p_*(*t*) and *n_d_*(*t*) are time-dependent densities of NPCs and PMCs, respectively; α(*t*) is the rate of NPC division; and *p*_i_ (*i* = 1–4) is the probability of NPC division modes, *p*_1_-*p*_4_ (see Fig. 1*B*). The total probability across all possible division/conversion modes is normalized by setting*: p*_1_ + *p*_2_ + *p*_3_ + *p*_4_ = 1. The factor 2 in front of the mode probability *p*_3_ reflects a production of 2 PMCs (see Fig. 1*B*). The parameters τ*_p_* and τ*_d_* represent the average lifespan of NPCs and PMC, respectively, in case when no cell division occurs. The meaning and values of τ can differ depending on whether the niche or the entire brain structure is analyzed. For example, for a niche, τ*_d_* reflects both the cell death and migration, whereas for the whole structure, τ*_dC_* includes only cell death. *Q_p_*(*t*) and *Q_d_*(*t*) are the rates of division/conversion of NSCs that result in either NPCs or PMCs, respectively.

Based on these definitions, the source functions *Q_p_*(*t*) and *Q_d_*(*t*) are expressed as follows:

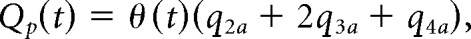


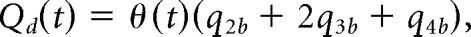
 where θ(*t*) is the time-dependent rate of NSC division and *q_j_* (*j* = 1–7) are the probabilities of all the NSC division modes, *q*_1_-*q*_4b_ (see Fig. 1*A*).

This system of kinetic equations (Eqs. 1 and 2) can be integrated for arbitrary time-dependent functions, α(*t*) and *Q_p_*(*t*), and specified initial conditions as follows:

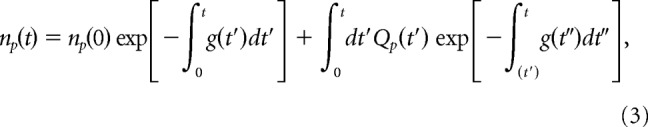
 where *g*(*t*) = α(*t*)(*p*_3_ + *p*_4_ − *p*_1_) + 1/τ*_p_*, and *n_p_*(0) is the NPC density at the initial moment *t*_0_ = 0. In Equation 3, NSC and NPC divisions are considered as two sources of NPCs.

The time-dependent density of PMCs is derived by integrating Equation 2 as follows:

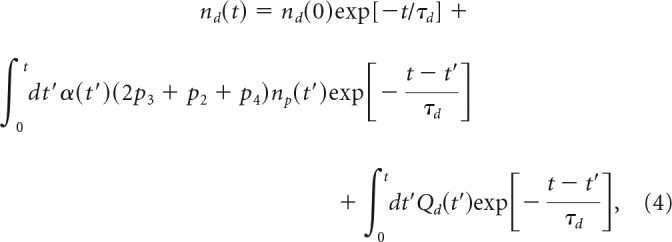
 where *n_d_*(0) is the PMC density at *t*_0_ = 0, and the NPC density *n_p_*(*t*) is the time-dependent function given by the analytical expression in Equation 3.

##### Periodic cell division.

The cell densities *n_p_*(*t*) and *n_d_*(*t*) can be periodic functions, if the α(*t*) and *Q_p_*_,_*_q_*(*t*) are periodic: α(*t*) = α(*t* + *T*); *Q_p,q_*(*t*) = *Q_p,q_*(*t* + *T*) for any arbitrary *t*, where *T* is the period. The *T* = 24 h was documented for cell replication in zebrafish neurogenic niches (Akle et al., 2017). However, the cell labeling (e.g., with BrdU) occurs at a specific phase of the process (*t*_0_ = 0), and the starting conditions for labeled cells are reflected in their initial densities *n_p_*(0) and *n_d_*(0). The general solutions of Equations 3 and 4 for labeled cells are nonperiodic functions because of these specific initial conditions. The expression for the cell densities in Equations 3 and 4 is simplified if probabilities of all division modes, *p_i_* and *q_j_*, are constant, and the probability of the conversion modes is small as follows:

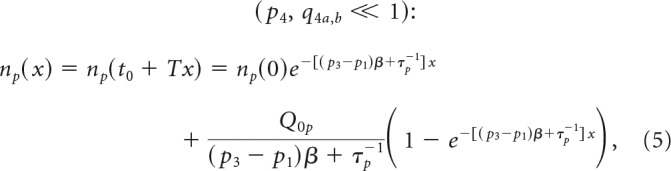


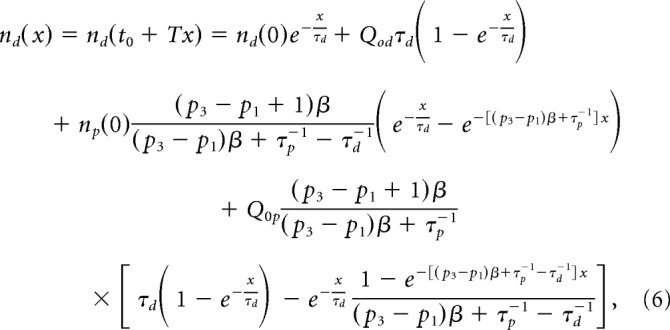
 where *x* = *t/T* is the number of days since BrdU labeling, *n_p_*(0) and *n_d_*(0) are NPC and PMC densities at the moment of labeling, respectively, and β and *Q*_0_ are the numbers of NPC and NSC divisions for the period *T* (in our case, daily): β = $\int_{0}^{T}\alpha \left(t\right)dt$ and *Q*_0_ = $\int_{0}^{T}\theta \left(t\right)dt$. The values *Q_op_* = *Q*_0_(*q*_2*a*_ + 2*q*_3*a*_), and *Q_od_* = *Q*_0_(*q*_2*b*_ + 2*q*_3*b*_) are daily production of NPCs and PMCs by NSCs. Equation 5 shows that the overall (total) lifespan for NPCs, τ*_p_^tot^*, depends on a combination of attrition through cell division 1̸(*p*_3_ − *p*_1_)β and cell death τ*_p_*, and can be calculated as follows:

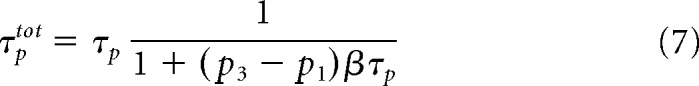
 with β = 1 (Akle et al., 2017).

##### Kinetics of labeled cells.

The derived expressions for *n_p_*(*x*) and *n_d_*(*x*) provide analytical formulas for the densities of labeled cells (e.g., BrdU- and EdU-positive) and colabeled cells, *n_B_*(*x*), *n_E_*(*x*), and *n_C_*(*x*), respectively, as follows:

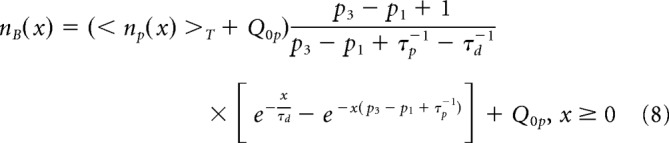


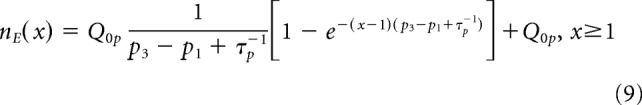


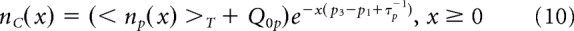
 where < *n_p_*(*x*) >*_T_* = *Q*_0*p*_/(*p*_3_ − *p*_1_ + τ_*p*_^−1^) is the number of NPCs averaged over the period *T*. < *n_p_*(*x*) >*_T_* can be also considered as a steady-state NPC density, if the cell division rates α(*t*) and θ(*t*) are time-independent constants. Equations 8–10 are valid in case the division of NSCs produces only NPCs; thus, *Q*_0_*_p_* = *Q*_0_.

##### Model analysis of experimental data.

The analytical formulas from Equations 8–10 were used in the parametric analysis of the experimental data on cell kinetics in the zebrafish cerebellar neurogenic niche and the entire cerebellum. The values of model parameters in the kinetics equations were inferred from the comparison between theoretical and experimental results. We determined four independent parameters from which other values could be derived: (1) *Q*_0_, the number of active NSCs per day; (2) γ*_p_* = $\frac{1}{\tau_{p}^{tot}}$ = (*p*_3_ − *p*_1_) + τ_*p*_^−1^, the daily rate of NPC attrition due to cell division and cell death; (3) γ*_d_* = (2*p*_3_ + *p*_2_) = (1 + *p*_3_ − *p*_1_), the daily rate of PMC production; and (4) τ*_dC_*, PMC lifespan in the whole cerebellum based on cell death only, or τ*_dN_*, PMC lifespan in the niche, including time of migration and cell death. These four parameters describe experimental data represented by 9 data points in Control or 15 data points in HCI.

The model does not provide values for specific probabilities of division modes but infers the value for their differences (e.g., *p*_3_ − *p*_1_), responsible for the growth or decline of the NPC pool. The mode *p*_2_ of NPC divisions with the probability *p*_2_ = 1 − *p*_3_ − *p*_1_ does not affect NPC density, and *p*_2_ cannot be independently determined from our experimental data.

Inferred independent parameters can be used to determine other characteristics of the cell kinetics, such as the daily averaged number of NPCs for the whole cerebellum < *n_p_* >*_tot_* = $\frac{Q_{0}}{\gamma_{p}}$ or the characteristic time τ*_p_* = $\frac{1}{1+\gamma_{p}-\gamma_{d}}$ of NPC death or migration. Model analysis of the cerebellar niche data yields an average NPC number in the niche < *n_p_* >*_niche_*. The model infers the PMC lifespan in the whole cerebellum, τ*_dC_*, in days, their daily average number < *n_d_* >*_tot_* = $\frac{\gamma_{d}Q_{0}}{\gamma_{p}}\tau_{dC}$, and the number of migrating PMCs, daily < *n_d_* >*_migr_* ≅ γ*_d_* < *n_p_* >*_niche_* − < *n_d_* >*_niche_*.

The relative abundances for BrdU-positive, EdU-positive, or colabeled cells were calculated, with their sum normalized to 100%. These relative abundances were determined for each fish, and then mean values and SDs were computed for the entire group (feeding condition by day), as presented in Results.

## Results

### The 2D and 3D evaluation of adult neurogenesis in zebrafish

**Figure 2.**
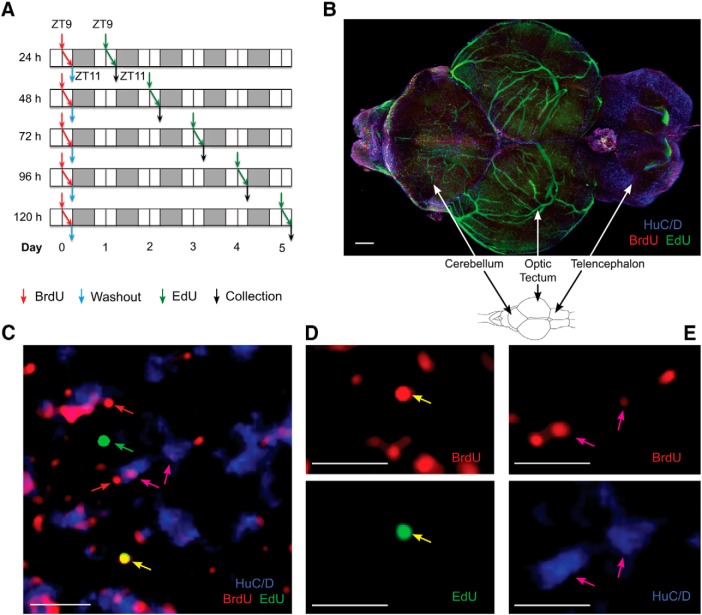
The pulse-chase BrdU-EdU protocol reveals proliferating cells and newly developing neurons in young adult zebrafish brain. ***A***, Experimental protocol involving BrdU pulse and EdU chase 1–5 d thereafter. Arrows indicate BrdU administration (red), BrdU washout (blue), EdU injection (green), and sample collection (black). BrdU and EdU exposure at ZT9–11, with ZT0 = lights-on time; 14:10 light-dark cycle. White area represents light. Gray area represents dark. *N* = 5 or 6 fish per time point. ***B***, Cleared zebrafish brain following a 15 d pulse-chase. Scale bar, 150 μm. ***C***, Labeled cells within cerebellar neurogenic niche, following 15 d chase. Arrows indicate BrdU/EdU colocalization (yellow), EdU-only (green), BrdU-only (red), and BrdU/HuC/D colocalization (magenta). Scale bar, 10 μm. ***D***, Single-channel images for the colabeled yellow stem cell in ***C***, BrdU (red) and EdU (green) representing NSCs. ***E***, Single-channel images for cells colabeled for BrdU (red) and HuC/D (blue), representing immature neurons. Scale bar, 10 μm.

**Figure 3.**
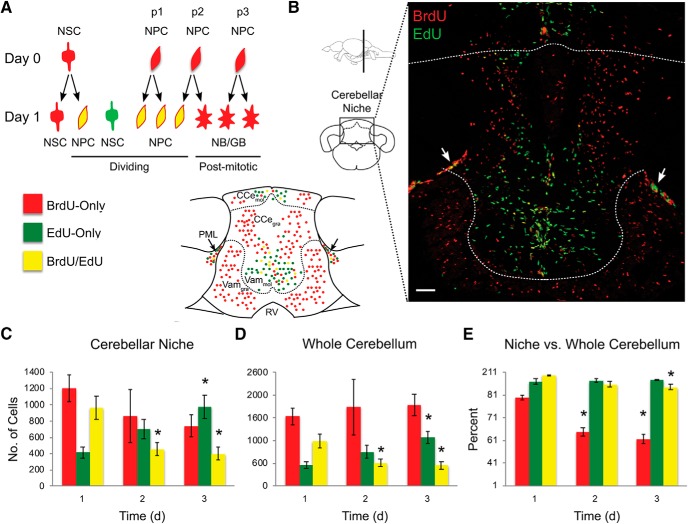
Daily proliferative activity in the brain of normal 1-year-old zebrafish. Cell proliferation in the cerebellum over a 1 to 3 d BrdU/EdU pulse-chase. ***A***, Schematics of representative cell division modes and cell color on the day of BrdU pulse (day 0) and EdU chase (day 1). RV, Rhombencephalic ventricle. ***B***, Representative image of labeled cells in the cerebellar niche and outside parenchyma on day 3 of BrdU/EdU pulse-chase, and corresponding schematics. Arrows indicate posterior mesencephalic layer (PML). Scale bar, 50 μm. ***C***, Number of labeled cells in the cerebellar neurogenic niche. ***D***, Number of labeled cells in the whole cerebellum. ***E***, Percentage of labeled cells in the neurogenic niche versus whole cerebellum. Cell counts conducted in brain sections. Red represents BrdU-only. Green represents EdU-only. Yellow represents BrdU/EdU colocalized. *N* = 5 or 6 fish per time point. Data are mean ± SEM. *p < 0.05, relative to day 1.

The investigation into the kinetics of cell replication and migration, and acquisition of neuronal fate in adult zebrafish brain considered the known types of cells involved in adult neurogenesis, NSCs and NPCs, and their potential ways of division or conversion (Fig. 1*A*,*B*). Two different S-phase markers, BrdU and EdU, and a pulse-chase paradigm were used, with an interval between the introduction of the two markers varying between groups of fish (Fig. 2*A*). The exposures were conducted at the time of peak proliferation in the brain of adult zebrafish, ZT9–11 (Akle et al., 2017). Fish were collected at the end of the 2 h EdU exposure. Brains were processed using two immunohistochemical techniques: a novel CLARITY approach, which provided a comprehensive 3D view of all the 16 neurogenic niches (Fig. 2*B*; Movie 1), and a traditional 2D brain coronal section technique (Fig. 3*B*). Both approaches allowed quantification of three types of cells: those labeled with BrdU-only, EdU-only, or colocalized BrdU and EdU (Figs. 2*C*,*D*, 3*B*; Movies 1, 2). A 15 d pulse-chase also revealed cells colabeled for the neuronal marker HuC/D and BrdU, indicating that some of the postmitotic progeny of cells undergoing division on day 0 acquired neuronal fate (Fig. 2*C*,*E*).

Movie 1.The 3D confocal imaging of proliferative activity within a whole cleared adult zebrafish brain. Following a 15 d pulse-chase protocol: BrdU-only (red) within neurogenic niches, NSCs dividing on day 0 but not day 15; BrdU-only (red) outside neurogenic niches, postmitotic cells that migrated following day 0 division cycle; EdU-only (green), NSCs and NPCs undergoing division on day 15, but not on day 0; EdU+BrdU (yellow), NSCs dividing on both day 0 and day 15; HuC/D-only, neurons; and HuC/D+BrdU (magenta), postmitotic cells of day 0 acquiring neuronal fate. Note green autofluorescence in the vasculature, especially prominent in the optic tectum.10.1523/JNEUROSCI.2730-18.2019.video.1
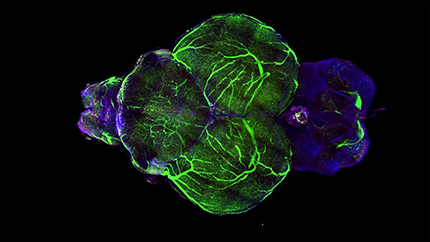

Movie 2.3D automated counting of a cleared whole cerebellum in a HCI adult zebrafish. Following a 15 d pulse-chase protocol in an HCI fish, EdU^+^ cells (green, left) in the cerebellum were quantified (white, right) in the *z* stack (8 frames/s) using the 3D object counter in Fiji. Fiji software was also used to reduce background and vasculature autofluorescence. False-positive counts were eliminated based on voxel volumes (pixel3).10.1523/JNEUROSCI.2730-18.2019.video.2
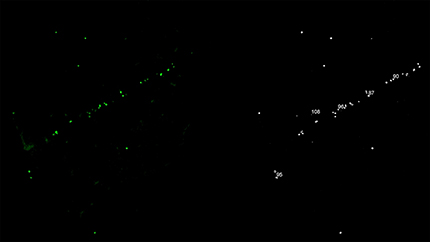

The distribution of cells in the neurogenic niches and in the outside brain parenchyma reflected the presence of both proliferating and PMCs (Fig. 3*A*). The BrdU-only cells staying in the niche represented, in part, NSCs that were undergoing S-phase on day 0, but not on the consecutive days. The rest of the BrdU-only cells were postmitotic progeny of those NSCs and NPCs that were dividing on day 0. Accordingly, by day 3, the BrdU-only cells included the NSCs of day 0 and PMCs created by the day 0 NPCs over the 3 consecutive days, days 0–2. Those PMCs could be observed in either the niche or the outside cerebellar parenchyma to which they have migrated (Fig. 3*B*). The EdU-only cells represented those NSCs and NPCs that were undergoing S-phase on the day of sample collection. They were present almost exclusively in the neurogenic niches, as was expected based on a short 2 h postexposure survival of the animals. Nevertheless, some EdU-only cells were found right outside the border of the molecular cell layer and, though very rarely, further away from the niche. These cells could represent migrating transient progenitors, including the proliferating neuroblasts described previously (Kaslin et al., 2009).

The colabeled BrdU/EdU cells, those that have undergone S-phase on both day 0 and the day of EdU treatment, were present in the neurogenic niches (Figs. 2*C*, 3*B*; Movie 1). In view of a reported relatively long interval between consecutive divisions of the same NSC (Chapouton et al., 2010; Rothenaigner et al., 2011; Barbosa et al., 2015) (i.e., at least longer than 5 d), the colabeled cells of the up to 5 d chase were considered to represent NPCs, cells that can undergo several consecutive divisions (Fig. 3*A*). This was in contrast to similarly colabeled cells following a 15 d chase. A long interval between BrdU and EdU exposure excluded a possibility of day 0 NPCs still cycling, strongly suggesting that the colabeled cells are NSCs that reentered S-phase after a 15 d quiescence (Fig. 2*D*).

### Proliferative activity in a neurogenic niche of a young mature zebrafish

The quantitative analysis was conducted in the cerebellar niche (Fig. 3*B*; Movie 1), due to its well-delineated anatomy and active proliferative activity (Zupanc et al., 2005; Kaslin et al., 2009, 2013) that follows a robust circadian pattern (Akle et al., 2017). The analysis of the raw data collected in Control animals over a 3 day period revealed high interindividual difference in their proliferative capacity (Fig. 3*C*,*D*). Despite this, there was a remarkable similarity among individuals in the relative cell abundances (i.e., when the number of labeled cells inside individual's niche was compared with that in the whole cerebellum) (Fig. 3*E*). As a result, in the neurogenic niche, the significant decline in the abundance of BrdU-only cells on days 2 and 3 became evident (Fig. 3*E*; day 1, 77.7 ± 1.7; day 2, 47.1 ± 3.7; day 3, 41.2 ± 3.8; *t*_(4)_ = 7.56, *p* = 1.6e-3 and *t*_(4)_ = 8.69, *p* = 9.7e-4, day 1 vs 2 and day 1 vs day 3, respectively, unpaired *t* test). This change reflected interplay between the creation of new PMCs (progeny of NSCs and NPCs that were undergoing S-phase on day 0), PMC migration from the niche, and some cell death. Low interindividual variation in relative cell abundances suggested that the Control fish had major similarities in the rate of cell renewal, migration and cell survival in the cerebellum, independent of the proliferative capacity of an individual.

There was also a decline in the number of the colabeled BrdU/EdU cells in the niche and in the whole cerebellum (Fig. 3*C*,*D*). This was consistent with gradual elimination of transient amplifying cells, NPCs of day 0, through terminal division or cell death. In contrast, we found a significant increase in EdU-only cells over this period of observation (Fig. 3*E*; day 1, 452.7 ± 71.2; day 2, 756.6 ± 147.7; day 3, 1071.3 ± 132.6; *t*_(4)_ = −2.07, *p* = 0.11 and *t*_(4)_ = −4.11, *p* = 0.01, day 1 vs day 2 and day 1 vs day 3, respectively, unpaired *t* test). On day 1 after BrdU, the EdU-only cells corresponded only to NSCs that were undergoing DNA replication on that very day (Fig. 3*A*). This is because the cells that were dividing on day 0 would either remain quiescent (NSCs) or, if those were NPCs repeating the cycle the next day, would carry both labels. However, beyond day 1, EdU-only cells included both new NSCs dividing on that day, as well as still cycling NPCs formed over the previous days (but not on day 0, which would contribute a BrdU label). For example, if NPC resulted from a division of NSC on day 1, it would not carry BrdU, but would be likely to continue cycling on day 2 or day 3, thus picking up EdU administered on one of those subsequent days. Together, gradual increase in the number of such EdU-only cells was consistent with the replacement of the expiring day 0 NPCs by new active NPCs, and a new cohort of active NSCs initiating cell cycle on a given day. The observed balance between these distinct populations of labeled cells corresponded to a day-to-day consistency of cell proliferation and migration, also confirmed by the similar number of EdU-positive cells present in the healthy cerebellar niche on each day (mean ± SEM, 1304 ± 72 cells).

### Proliferative activity in adult brain is altered by chronic HCI

We previously reported that animals exposed to HCI diet since young age demonstrate premature aging phenotype and reduced proliferation in the brain compared with age-matched control (Stankiewicz et al., 2017). Here, we further explored whether this phenomenon is limited to the absolute amount of proliferation in the HCI brain or may also involve changes in the kinetics of cell proliferation and migration. To exclude a potential acute effect of HCI on cell proliferation, these 1-year-old animals were maintained on normal control diet for 2 months before the experimental procedures described here.

**Figure 4.**
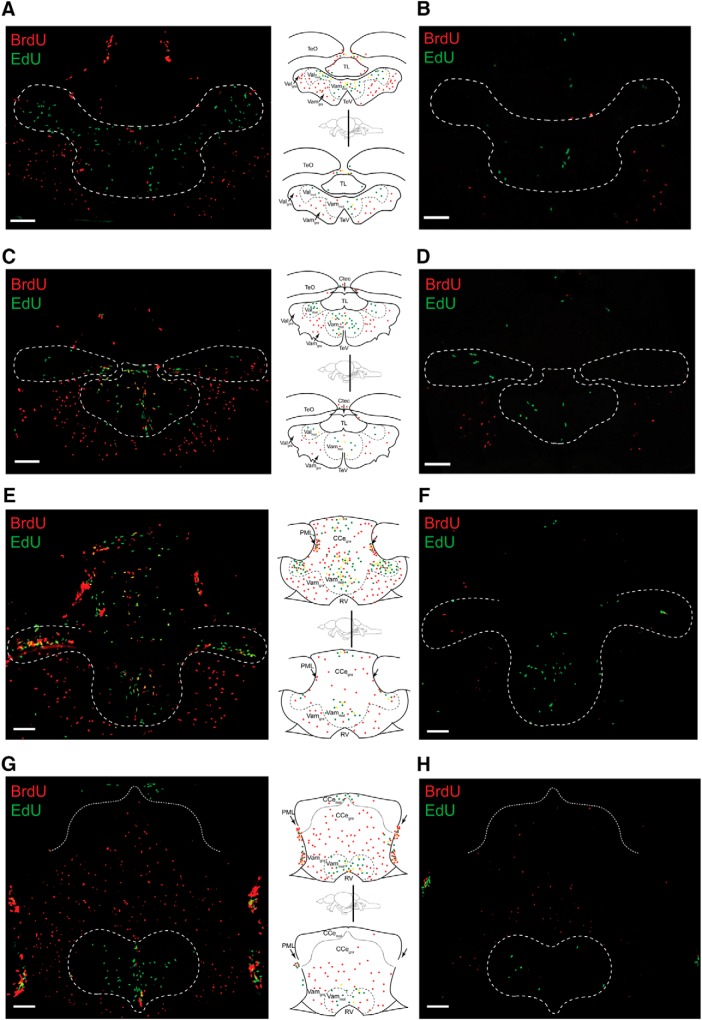
Chronic HCI alters proliferative capacity of zebrafish brain. Representative cerebellar images and corresponding schematics in 1-year-old Control (left column, top) and HCI (right column, bottom) zebrafish on day 3 of BrdU-EdU pulse-chase. ***A***, ***B***, Rostral valvula cerebelli (corresponding to Wulliman et al., 1996, atlas section 179). ***C***, ***D***, Valvula cerebelli (corresponding to Wulliman et al., 1996, atlas section 196). ***E***, ***F***, Caudal valvula cerebelli and rostral corpus cerebelli (corresponding to Wulliman et al., 1996, atlas sections 201–204). ***G***, ***H***, End of valvula cerebelli and mid corpus cerebelli (corresponding to Wulliman et al., 1996, atlas sections 213–219). TeO, Optic tectum; Ctec, commissura tecti; TeV, tectal ventricle; PML, posterior mesencephalic layer; RV, rhombencephalic ventricle; TL, torus longitudinalis. Dashed line indicates neurogenic cerebellar niche regions. Red represents BrdU-only. Green represents EdU-only. Yellow represents BrdU/EdU colocalized. Scale bar, 50 μm.

**Figure 5.**
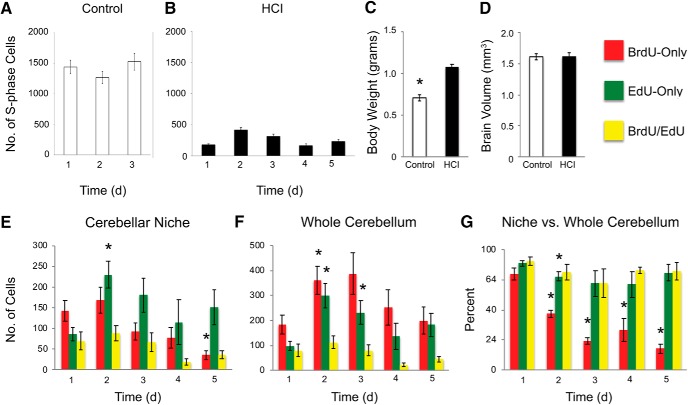
Daily proliferative activity in the brain of 1-year-old zebrafish with chronic HCI. Quantitative evaluation of cell proliferation in the cerebellum over a 1–5 d of BrdU-EdU pulse-chase. ***A***, ***B***, Number of cells in S-phase on consecutive days, at daily peak time (following 2 h EdU exposure at ZT 9–11) in Control (***A***) and HCI (***B***) fish. ***C***, Body weight (grams) in Control (white) and HCI (black) fish. ***D***, Brain volume (mm^3^) in Control (white) and HCI (black) fish. ***E***, Number of labeled cells in the cerebellar neurogenic niche. ***F***, Number of labeled cells in the whole cerebellum. ***G***, Percentage of labeled cells in the neurogenic niche versus whole cerebellum. Cell counts conducted in brain sections. Red represents BrdU-only. Green represents EdU-only. Yellow represents BrdU/EdU colocalized. *N* = 5 or 6 fish per time point. Data are mean ± SEM. *p < 0.05, relative to day 1.

The HCI resulted in reduced proliferation in all 16 neurogenic niches, and this was consistent throughout the individual neurogenic niche, as illustrated in Figure 4 on multiple cerebellar planes. Due to the increased day-to-day variability in the number of cells in S-phase in HCI, when compared with Control (Fig. 5*A*,*B*), we increased the pulse-chase interval up to 5 d, to improve the cell kinetics analysis. Notably, whereas the body weight was increased in HCI animals, their brain volume remained similar to that in Control (Fig. 5*C*,*D*).

The quantitative analysis of the HCI cerebellar niche demonstrated that, over the period of chase, relative to day 1, there was a decline in the number of BrdU-only and colabeled BrdU/EdU cells, and an increase in the number of EdU-only cells (Fig. 5*E*). There was also a major reduction in the number of the BrdU-only cells in the neurogenic niche over a 5 d period compared with the whole cerebellum, reflecting migration of PMCs out of the niche (Fig. 5*G*). This dynamic was similar to that observed in Control fish. However, HCI animals appeared to lack the orderly day-to-day progression of cell proliferation observed in Control zebrafish brain. Understanding such complex dynamic datasets requires mathematical model analysis.

### Mathematical model analysis of the cell kinetics of normal and HCI neurogenic niche

The experimental techniques allow us to directly observe the abundance of cells that were undergoing S-phase at the time of a pulse, chase, or both. From such observations, we would like to infer the number of certain cell types, the probability of different modes of division, the rate of migration or cell death, and other kinetic properties of a neurogenic niche and the brain structure as a whole. To do so, we developed a mathematical model that ties together the labeling frequency with cell kinetics. The model is presented in detail in Materials and Methods and, in general formulation, includes all cell division modes and potential fates for NSCs and NPCs (Fig. 1*A*,*B*). However, the goal of any mathematical model is to describe the data and make accurate predictions on the basis of a minimal number of independent parameters. Thus, based on the earlier descriptions of adult neurogenesis in zebrafish and other model organisms (Bonaguidi et al., 2011; Encinas et al., 2011; Rothenaigner et al., 2011; Ponti et al., 2013; Barbosa et al., 2015; Calzolari et al., 2015; Obernier et al., 2018; Silva-Vargas et al., 2018), we accepted the following assumptions. The NSCs divide infrequently and asymmetrically (q2a, Fig. 1*A*). The NPCs undergo several consecutive cell cycles, with variable probability for each division mode, conversion, or cell death (Figs. 1*B*, 2*A*).

**Figure 6.**
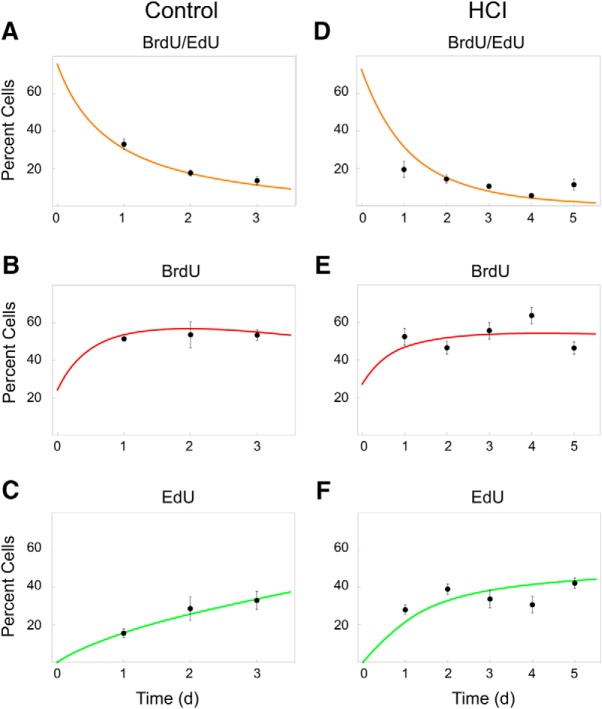
The kinetics of cell proliferation in adult cerebellum under normal and accelerated aging conditions. Comparison between the model predictions and the experimental data in Control zebrafish (***A–C***) and HCI animals with accelerated aging (***D–F***). ***A***, ***D***, Colocalized BrdU/EdU cells: NPCs. ***B***, ***E***, BrdU-only cells: stem cells active during initial pulse (day 0) and PMCs that are progeny of cells replicating on day 0. ***C***, ***F***, EdU-only cells that are in S-phase on the day of chase and sample collection (days 1–5). *y* axis indicates percentage cells with specific label in the whole cerebellum.

To evaluate the accuracy of the model, we fit the four independent model parameters (see Materials and Methods) based on a fraction of the experimental data in Control fish, and validated the model performance on the remaining data points. The model demonstrated excellent fit to the BrdU-only, EdU-only, and colabeled cell frequencies in the raw data in Control animals, suggesting that it was able to capture cell kinetics with high precision. Figure 6*A–C* illustrates the relative time-dependent abundancies of the labeled cells in the whole cerebellum. The fit of the model curves to the experimental data supported our assumption that, on average, one NSC division leads to the production of one NSC and one NPC in the cerebellum of the Control fish. This was consistent with either the predominant asymmetric mode of NSC division (q2a, Fig. 1*A*) or alternatives that, on average, could yield the similar number of NSCs and NPCs. For example, a scenario where half of NSCs divided symmetrically to produce two NSCs, while the other half divided to produce two NPSs, led to the similarly good fit of the model curves. However, when other variable combinations of NSC division modes were tested, the model curves significantly deviated from the best fit presented in Figure 6.

The model provided the following quantitative description of the system and its behavior (Table 1). In healthy 1-year-old Control zebrafish, the daily average number of active NPCs present in the whole cerebellum was predicted to be more than twice higher than the number of active NSCs (964 vs 453 cells, respectively). This supported the notion that a large number of NPCs repeat the cycle over several days after an initial labeling with BrdU on day 0. Indeed, an average overall lifespan of NPC was predicted to be 2.1 d. The loss of NPCs was largely explained by depletion through division (−241 cells per day), as a result of a 30% higher chance of a symmetric cell division generating two postmitotic progeny cells (*p*_3_), relative to a self-renewal division (*p*_1_) creating two NPCs (Fig. 1*B*). In contrast, if NPCs would not divide, their lifespan was predicted to be 4.5 d. The average daily gain in postmitotic progeny (i.e., production − cell death) was predicted to be 1205 cells, with only 726 PMCs remaining inside the niche on any given day, and with characteristic migration time of 0.6 d (Table 1).

**Table 1.** Model-derived characteristics of cell kinetics in the cerebellar neurogenic niche of Control zebrafish and HCI animals with accelerated aging due to HCI

Parameter	Control	HCI
NSCs in S-phase, daily no.	453	73
NPCs in S-phase, daily no.	964	121
NPC overall lifespan, days	2.1	1.7
NPC loss/gain through division, daily	(−)241	(+)49
NPC cell death/migration, days	4.5	1
PMCs produced, daily no.	1205	74
PMCs in niche, daily no.	726	120
PMCs migrate outside niche, days	0.6	2.2

In HCI brains, the mathematical model revealed a major distortion in cell kinetics. High day-to-day variability in proliferation within the HCI fish cerebellum (Fig. 5*B*) inevitably led to a less accurate fit of the model curves to the experimental data (Fig. 6*D–F*), compared with the fit attained for Control (Fig. 6*A–C*). The number of cycling NSCs and NPCs was predicted to be ∼6 and 8 times lower, respectively, relative to Control (Table 1). Moreover, unlike NPCs in Control favoring production of postmitotic daughter cells (*p*_3_ > *p*_1_), in HCI, NPCs were predicted to favor self-renewal divisions (*p*_1_ > *p*_3_). The analysis estimated a 40% increase in the mode of division producing two NPCs (*p*_1_) over that leading to two PMCs in HCI (*p*_3_). This caused a daily gain in NPCs in the HCI neurogenic niche (49 cells per day), unlike a daily loss of NPCs in Control (Table 1). However, despite such relative overproduction of NPCs, the overall lifespan of these HCI cells was predicted to be shorter than in Control (1.7 vs 2.1 d, respectively). This was a result of an increase in the rate of NPC death. The model predicted that, if NPCs would not be, in part, depleted through cell division, they would survive, on average, for 1 d in HCI animals, compared with 4.5 d in control fish (Table 1). A greater survival of NPCs in HCI fish through division mode, versus cell death, further reflected the relative overproduction of NPCs in HCI brains. Notably, the HCI animals had slower migration of PMCs from the neurogenic niche (2.2 d vs 0.6 d in Control; Table 1).

## Discussion

Accurate quantitative description of the cell kinetics within adult brain is essential for understanding normal age-related changes in neurogenesis, and for predicting long-term effects of internal and environmental factors on this important process. Here we reveal orderly daily cell kinetics within a neurogenic niche of a healthy adult vertebrate and major changes that result from chronic HCI, a factor known to accelerate aging of cognitive functions and organism as a whole (Poulose et al., 2017; Stankiewicz et al., 2017). Our relatively high-throughput experimental techniques and mathematical model can accurately describe the cell kinetics within a large cell population, providing the necessary quantitative approach to early detection of altered neurogenesis. This opens new opportunities for quantitative evaluations of the efficacy of prophylactic and therapeutic agents within a neurogenic niche, and for designing drugs targeting specific type of cells at different ages or certain stages of disease.

### The high-throughput approach to adult neurogenesis

Our understanding of the normal mechanisms of adult neurogenesis in vertebrates, along with age-pathology relationships and drug-induced modifications, could significantly benefit from high throughput methods in whole animal models. The zebrafish model already benefited high-throughput needs of developmental biology, genetics, and drug screens (Holtzman et al., 2016), and its highly active neurogenesis is yet another well-appreciated advantage (Zupanc et al., 2005; Grandel et al., 2006; Kaslin et al., 2009). Our approach, relying on the circadian pattern of adult neurogenesis in zebrafish (Akle et al., 2017), allows for documenting cell kinetics and cell fate in all 16 neurogenic niches of the zebrafish brain using 2D and 3D visualization, with automatic quantification of proliferation rate, cell fate, and paths of migration. This also provides for effective investigation into the role of the neurogenic niche milieu in neurogenesis, especially important in view of adult neurogenesis in zebrafish actively responding to aging, diet, toxins, or other environmental factors that can modulate niche environment (Arslan-Ergul et al., 2013, 2016; Edelmann et al., 2013; Skaggs et al., 2014).

The complexity of the neurogenic process, with varying probability of diverse cell division or conversion modes, and variable survival or speed of migration of their progeny toward active neuronal networks, requires extensive mathematical analysis. Our model of cell kinetics in a neurogenic niche is developed based on a comprehensive set of independent parameters for proliferating stem cells and transient amplifying cells. It takes into account all their modes of division or conversion (Fig. 1), and can be used to analyze data collected using different experimental approaches and techniques. The model allows inference of the time-dependent densities of labeled and unlabeled cells, their cell types, rate of production, prevailing modes of division, speed of migration, and lifespan.

### Normal cell kinetics in the neurogenic cerebellar niche of young adults

In healthy young adults, we find highly active cell proliferation present in all 16 neurogenic niches of the brain. For cell kinetics analysis, we focused on the cerebellum, a structure that is well conserved between fish and mammals, and with abundant life-long neurogenesis in two principal proliferation zones (Zupanc et al., 2005; Grandel et al., 2006; Kaslin et al., 2009).

The perfect fit between the model curves and the experimental data in Control validates our model and assumptions made. The number of days that NPCs survive according to the model (2.1 d) is consistent with 2–3 consecutive divisions for transient amplifying cells reported earlier based on live imaging (Rothenaigner et al., 2011; Barbosa et al., 2015). Moreover, we find that, in normal fish, NPCs favor division into two daughter cells over that into two NPCs. The resulting PMCs expediently migrate from the niche to the surrounding brain parenchyma, with some demonstrating a newly acquired neuronal fate.

### Chronic HCI is detrimental to adult neurogenesis

The HCI leads to low levels of active NSCs. This may result from increased NSC quiescence or their loss. The model predictions can be further strengthened by the use of additional markers characteristic of quiescent NSCs, apoptosis, or those predictive of differentiation states (Rothenaigner et al., 2011; Barbosa et al., 2015; Aggarwal et al., 2016). Also, unlike typical asymmetric division (q2a, Fig. 1*A*), which creates NPC and maintains NSC pool (Kaslin et al., 2009; Mineyeva et al., 2018), a direct division or conversion into PMCs can cause NSC depletion (Barbosa et al., 2015). The latter, however, is unlikely in HCI fish because no increase in PMC numbers is observed.

The model also indicates that there is a relative overamplification of the NPC pool in HCI animals, despite their low absolute numbers. The model explains this by the higher probability of NPC division into two NPCs (*p*_1_ mode) than their division into two PMCs (*p*_3_). Such self-amplification of NPC pool is a common strategy documented in mammalian (Jacob and Osato, 2009; Kriegstein and Alvarez-Buylla, 2009) and zebrafish (Barbosa and Ninkovic 2016) brain for replenishing cells lost to injury. The observed relative overproduction of NPCs can be a compensatory response to their short lifespan in HCI brains, showing a much higher rate of cell death than in Control.

Another notable feature of HCI brain is slow migration of PMCs outside the niche, taking over 48 h, compared with ∼14 h for Control. This might reflect a pathologically slow migratory potential of these PMCs or significant changes in the niche milieu. Interestingly, in both the normal and HCI cerebellum, we observe replicating cells in the granular layer, outside the neurogenic niche. Their number is very small in normal animals but can rise to up to 30% in HCI. These cells might represent migrating proliferating neuroblasts and glioblasts, reported previously (Kaslin et al., 2009). An increase in the proportion of such cells in HCI could reflect yet another compensatory mechanism, with paucity of stem cells being compensated via modified kinetics of their progeny.

Our model allows for estimating an average lifespan for PMCs, if the total number of cells in the brain remains relatively constant over time. In case the cell numbers deviate significantly due to, for example, growth or neurodegeneration, the equations would require an introduction of an additional parameter, describing the rate of such process. Interestingly, at 1 year of age, HCI fish have brain volume similar to that in Control, despite the major decline in proliferative activity in the brain and significantly higher body mass. We are yet to learn at which age chronic HCI starts interfering with neurogenesis. If this occurs close to 1 year of age, the effect on brain volume would require some time to reach significance. Moreover, brain volume depends on not only the absolute number of newly divided cells and the rate of apoptosis, but also on the size of neuronal and glial cells, the amount of extracellular matrix, or extracellular space. These outstanding issues are to be reconciled through further research.

### The aging of neurogenesis and metabolic abnormalities

The disorders of human aging typically develop gradually over many years. The zebrafish is an excellent vertebrate model of gradual aging (Kishi et al., 2009), with relatively long lifespan of up to 6 years, well-defined senescence phenotype (Gerhard and Cheng, 2002), and gradual decline in neurogenesis (Edelmann et al., 2013; Stankiewicz et al., 2017). The latter may underlie concurrent age-dependent changes in their sleep (Zhdanova, 2011), circadian rhythms (Zhdanova et al., 2008), endocrine functions (Tsai et al., 2007), cognitive performance (Yu et al., 2006), and anxiety levels (Kacprzak et al., 2017). In HCI fish, high mortality rate at a relatively young age is associated with other signs of accelerated aging in HCI fish, including early onset of sarcopenia, scoliosis, sleep abnormalities, and compromised circadian patterns of behavior (Stankiewicz et al., 2017), all of which also occur in aging humans.

The extent of adult neurogenesis and its role in humans are yet to be fully clarified (Winner and Winkler, 2015; Boldrini et al., 2018; Sorrells et al., 2018). However, studies in animal models suggest a major role of adult neurogenesis in brain maintenance and repair (Katsimpardi and Lledo, 2018). One of the most prominent factors affecting both adult neurogenesis and aging is nutrition (Poulose et al., 2017), with caloric excess enhancing aging (Balasubramanian et al., 2017; for review, see Liang et al., 2018). The nature of the underlying processes is poorly understood, and their contribution to neurodegenerative disorders remains elusive (Poulose et al., 2017; for review, see Gao et al., 2017). Our approach can facilitate the current development of the zebrafish model of neurodegenerative disorders and diabetes (Bhattarai et al., 2017; Dorsemans et al., 2017b; Koehler and Williams, 2018), and help elucidating the mechanisms involved in cognitive aging.

In conclusion, our experimental strategy and mathematical model permit elaborate analysis of pathological conditions that, similar to chronic HCI, are causing acute or long-term damage to the proliferative capacity of a neurogenic niche. These approaches applied to a high-throughput animal model open new possibilities for basic research and search for effective treatments affecting adult neurogenesis.
